# Validating childhood asthma in an epidemiological study using linked electronic patient records

**DOI:** 10.1136/bmjopen-2014-005345

**Published:** 2014-04-23

**Authors:** Rosaleen P Cornish, John Henderson, Andrew W Boyd, Raquel Granell, Tjeerd Van Staa, John Macleod

**Affiliations:** 1 School of Social and Community Medicine, University of Bristol, Bristol, UK; 2 Clinical Practice Research Datalink (CPRD), Medicines and Healthcare products Regulatory Agency, London, UK

**Keywords:** Epidemiology, Primary Care

## Abstract

**Objective:**

To investigate the performance of parent-reported data in identifying physician-confirmed asthma.

**Design and setting:**

Validation study using linkage between the Avon Longitudinal Study of Parents and Children (ALSPAC) and electronic patient records held within the General Practice Research Database (GPRD).

**Participants:**

Participants were those eligible to participate in ALSPAC who also had a record in the GPRD; this included 765 individuals, just under 4% of ALSPAC-eligible participants. The analysis was based on 141 participants with complete parent-reported asthma data.

**Primary and secondary outcome measures:**

The main GPRD outcome measure was whether a child had a diagnosis of asthma before they were nine. Parent-reported measures were doctor diagnosis of asthma (before mean age 7.5 years), various outcomes based on wheezing and breathlessness recorded longitudinally between 6 months and 8.5 years. Secondary outcomes were bronchial hyper-responsiveness (BHR), forced expiratory volume in 1 s/forced vital capacity ratio and skin prick test responses.

**Results:**

Among the 141 participants with complete parent-reported data, 26 (18%) had an asthma diagnosis before age nine. Using general practitioner (GP)-recorded asthma as the gold standard, the question ‘Has a doctor ever diagnosed your child with asthma?’ was both sensitive (88.5%) and specific (95.7%). ‘Ever wheezed’ had the highest sensitivity (100%) but low specificity (60%). More specific definitions were obtained by restricting to those who had wheezed on more than one occasion, experienced frequent wheeze and/or wheezed after the age of 3, but these measures had low sensitivities. BHR only identified 50% of those with a GP-recorded diagnosis.

**Conclusions:**

Parental reports of a doctor's diagnosis agree well with a GP-recorded diagnosis. High specificity for asthma can be achieved by using detailed wheezing questions, although these definitions are likely to exclude mild cases of asthma. Our study shows that linkage between observational studies and electronic patient records has the potential to enhance epidemiological research.

**Strengths and limitations of this study**

- We were able to successfully link data from the Avon Longitudinal Study of Parents and Children (ALSPAC) with the General Practice Research Database (GPRD).
- The richness of the ALSPAC data allowed us to explore a number of different epidemiological constructs of asthma definition in relation to a recorded physician diagnosis.
- We were able to compare the relative performance of objective measurements and lung function to that of parent-reported symptom data in identifying physician-confirmed asthma.
- Information on wheezing was collected longitudinally between ages 6 months and 8.5 years.
- The proportion of participants with complete self-reported data from ALSPAC was relatively small.

## Introduction

Asthma is difficult to measure in epidemiological studies because there are no definitive diagnostic criteria. The presence of recurrent respiratory symptoms, particularly wheezing and breathlessness, is central to its diagnosis in children. Bronchial hyper-responsiveness (BHR) is also a feature of asthma^1^ ^2^ but is not consistently present and can exist in the absence of clinical symptoms. Other measures of lung function may be normal during asymptomatic periods and can also be abnormal in the presence of other respiratory diseases.^1^ Thus, owing to the variable nature of asthma symptoms—both between and within individuals—its diagnosis remains a clinical one and clinical assessment is regarded as the best method for validating self-reported asthma data.^3^

However, clinical examination is costly and questionnaires are often preferred in epidemiological studies. Questions on wheezing, such as those used in the International Study of Asthma and Allergies in Childhood (ISAAC),^4^ have been shown to provide valid measures of the prevalence of asthma in children.^5–8^ Having said this, it is not possible to reliably distinguish between wheezing due to viral respiratory infections, which are common among young children, and wheezing due to asthma. In addition, mistaking other respiratory noises as wheezing can lead to over-reporting.^7^ ^9^ ^10^ Consequently, it has been suggested that questionnaires should be supplemented by measurements of airway responsiveness.^11^ However, several validation studies have shown that agreement between such measures and clinical asthma is poor.^3^

In the UK, childhood asthma is predominantly diagnosed and managed in primary care. Linkage to primary care records therefore provides a potential means to validate self-reported data. In this study, we have compared longitudinal data on wheezing and other respiratory outcomes with linked electronic patient records to investigate the performance of epidemiological data in identifying physician-confirmed asthma.

## Methods

### Subjects

Participants were those eligible to participate in the Avon Longitudinal Study of Parents and Children (ALSPAC) who also had a record in the General Practice Research Database (GPRD; now the Clinical Practice Research Datalink: http://www.cprd.com). The GPRD is an anonymised database of primary care records of around five million patients in the UK. ALSPAC has been described in detail previously.^12^ Briefly, just over 20 000 pregnant women living in and around Bristol, UK with due dates during 1991–1992 were eligible to take part; 15 247 pregnancies were recruited (15 390 fetuses, of which 14 701 were alive at 1 year) and these have been followed up since birth (the ALSPAC website has a searchable data dictionary; http://www.bris.ac.uk/alspac/researchers/data-access/data-dictionary/ describing all available data).

### Linkage between ALSPAC and the GPRD

Linkage between ALSPAC and the GPRD was conducted by the National Health Service Information Centre (NHS IC) as a trusted third party, using a methodology to preserve anonymity. They had previously linked ALSPAC participants to the NHS Central Register, with a 99% match rate^12^; this was carried out on the basis of NHS ID number, name, date of birth and postcode using deterministic linkage. With approval from the NHS National Information Governance Board (NIGB) Ethics and Confidentiality Committee, the NHS IC used this information to identify ALSPAC eligible individuals who also appeared in the GPRD; they then sent an anonymised linking dataset to be stored securely at the GPRD. ALSPAC and GPRD data for linked individuals were merged and analysed in a safe setting at the GPRD offices. As GPRD is anonymous and collected on an opt-out basis, and anonymity was preserved using the safeguards described above, this piece of research does not require consent above and beyond the consent obtained for participation in ALSPAC. However, ALSPAC has recently been collecting consent from participants, who are now adults, for ongoing participation in the study as well as consent to extract information from health and other administrative records, and any participants who withdrew from the study or did not agree to their health records being extracted were excluded from the linkage.

### ALSPAC data

When the children were, on average, 7.5 years old, mothers were asked (via a postal questionnaire) whether a doctor had ever diagnosed their child with asthma. Data on asthma symptoms (wheezing and breathlessness) were collected at 6 months and approximately every 12 months thereafter (at 18, 30, 42, 57, 69, 81, 91 and 103 months). As well as asking whether the child had wheezed, the mothers were asked whether their child had experienced ‘wheezing with whistling on the chest when (s)he breathed’ and, if so, on how many occasions. Symptom questions referred to the previous 12 months, apart from at 6 and 57 months, which referred to the past 6 and 15 months, respectively. A child was defined to have wheezed if the response to either question about wheezing was yes and not wheezed if the response to both was no. Frequent wheeze was defined as wheezing with whistling on the chest on at least three occasions during the past 12 months. During a clinic attended between 8 and 9 years, forced expiratory volume in 1 s (FEV_1_) and forced vital capacity (FVC) were measured by spirometry and bronchial responsiveness was measured using the rapid methacholine inhalation test^13^; the dose–response slope of FEV_1_ per µmol methacholine was used to classify participants into four categories: none, low, moderate and high, based on tertiles of the dose–response slope; BHR was defined as being the highest tertile. Sensitisation to house dust mite, cat and mixed grass was assessed by skin prick tests with a positive response defined as a mean weal diameter of 2 mm or greater; this definition identified over 90% of participants sensitised to any one of a panel of up to 12 allergens.^14^ Symptom data were converted to ‘ever wheezed’, ‘ever reported frequent wheeze’ and ‘ever had breathlessness’—all defined as positive if there was a positive response at any time point and negative if all responses were negative. Participants were classified according to whether or not their FEV_1_/FVC ratio was less than 85% and whether or not they had at least one positive skin prick test.

### GPRD data

Patients were defined as having a general practitioner (GP) diagnosis of asthma if they had a Read code (the clinical coding system used by GPs in the UK—http://www.connectingforhealth.nhs.uk/systemsandservices/data/uktc/readcodes/index_html) indicating an asthma diagnosis (any Read code starting with ‘H33’) in their record. In order to coincide with the timing of ALSPAC measurements, participants were classified according to whether they had a diagnosis before 9 years of age. Thus, participants who had no asthma diagnosis but left a GPRD practice before nine were excluded as it could not be determined whether they were subsequently diagnosed with asthma. Conversely, those who were not in the GPRD from birth were not excluded because historic diagnoses appeared in patients’ records.

### Statistical methods

Sensitivity, specificity and predictive values for ALSPAC outcomes and combinations of outcomes were calculated using GP-recorded diagnosis as the gold standard. Exact CIs were calculated based on binomial probabilities. These analyses were restricted to those with complete parent-reported data from ALSPAC. Because the question about a doctor's diagnosis of asthma was asked, on average, a year earlier than the last wheezing question as well as before lung function was measured, this outcome was also compared to GP-recorded asthma before age 8 years in order to evaluate the likely impact of this age gap on the estimated sensitivity and specificity. In order to try to restrict to current asthma, we looked at whether children with an early diagnosis had evidence of persistent asthma by school age or beyond. There was only one child in the complete-case analysis with an early diagnosis (aged 3) but no further evidence of asthma. However, since this child left the GPRD at age 6, it could not be determined whether or not they had persistent asthma and they were left in the analysis. Analyses were carried out using Stata V.12.0.

## Results

Of the live births linked by the NHS IC, 765 appeared in the GPRD (this constitutes 4% of those eligible to take part in ALSPAC); 4 individuals did not want their health records accessed and 1 registered into and left the GPRD on the same day. Of the remaining 760 individuals, 61 without an asthma diagnosis transferred out of the GPRD before the age of 9, leaving 699 participants with known asthma status. Of these, 488 (70%) had enrolled in ALSPAC, 251 (51%) had information on whether a doctor had ever diagnosed asthma, 141 (29%) had complete parent-reported asthma data; slightly fewer had BHR, FEV_1_, FVC and skin prick test data. Characteristics of all linked individuals with known asthma status and those included in the analysis are shown in table 1. Among the former, there were 115 children with a diagnosis of asthma before age 9, giving a cumulative incidence of 16%. This figure was slightly higher (18%) among those with complete ALSPAC data; these children were also less likely to live in deprived areas, as indicated by the index of multiple deprivation (IMD 2007; χ^2^=18.8, p=0.001).

**Table 1:** Characteristics of all linked participants with known asthma status compared to those with complete ALSPAC parent-reported data

GPRD-recorded characteristics	Linked participants who did not exit GPRD before age 9 (n=699)	Participants with complete ALSPAC parent-reported data* (n=141)
Sex—male	348 (50%)	70 (50%)
IMD^2^ quintile—least deprived	68 (13%)	24 (24%)
Second	155 (30%)	30 (30%)
Third	120 (24%)	27 (27%)
Fourth	58 (11%)	8 (8%)
Most deprived	109 (21%)	11 (11%)
Asthma—cumulative incidence	115 (16%)	26 (18%)

*The denominator for IMD is slightly lower (510 for all linked participants with GPRD asthma status at age 9; 100 for those with complete ALSPAC parent-reported data) as this variable is not complete within GPRD.

ALSPAC, Avon Longitudinal Study of Parents and Children; GPRD, General Practice Research Database; IMD, index of multiple deprivation.

Table 2 shows the proportions with and without a GP diagnosis correctly identified by different ALSPAC variables, as well as the overall percentage reporting each outcome. Wheezing was common: 51% of the children had ever wheezed and 27% reported frequent wheeze on at least one occasion.

**Table 2:** Cross tabulation of key ALSPAC asthma variables and GPRD asthma

ALSPAC asthma outcome	GP-recorded diagnosis of asthma		Overall
	No	Yes	
Doctor diagnosis of asthma by 7.5 years			
No	110 (95.7%)	3	113
Yes	5	23 (88.5%)	28 (19.9%)
Ever reported breathlessness in the past 12 months			
No	96 (83.5%)	6	102
Yes	19	20 (76.9%)	39 (27.7%)
Ever reported wheezing in the past 12 months			
No	69 (60.0%)	0	69
Yes	46	26 (100%)	72 (51.1%)
Wheezing reported on at least two occasions			
No	88 (76.5%)	3	91
Yes	27	23 (88.5%)	50 (35.5%)
Wheezing reported after the age of 3 years			
No	96 (83.5%)	9	105
Yes	19	17 (65.4%)	36 (25.5%)
Ever reported a frequent wheeze			
No	96 (83.5%)	7	103
Yes	19	19 (73.1%)	38 (27.0%)
Reported a frequent wheeze after the age of 3 years			
No	110 (95.7%)	11	121
Yes	5	15 (57.7%)	20 (14.2%)

ALSPAC, Avon Longitudinal Study of Parents and Children; GP, general practitioner; GPRD, General Practice Research Database.

Of the ALSPAC variables, ‘ever wheezed’ had the highest sensitivity (100%; 95% CI 86.8% to 100%) but low specificity (60%; 50.4% to 69.0%). The question ‘Has a doctor ever diagnosed your child with asthma?’ was both sensitive (88.5%; 69.8% to 97.6%) and specific (95.7%; 90.1% to 98.6%). By restricting to those who had wheezed on more than one occasion, experienced frequent wheeze or wheezed after the age of 3, more specific asthma definitions were obtained (table 2 and see online supplementary table S1). However, the consequent losses in sensitivity were substantial. Positive and negative predictive values are given in table 3. These confirm the above results. BHR only identified 50% of those with a GP diagnosis; the specificity was also relatively low (75%). The sensitivities of FEV_1_/FVC ratio and skin prick tests were also low (see online supplementary table S2).

**Table 3:** PPV and NPV of different asthma outcome measures

ALSPAC asthma outcome	PPV (95% CI)	NPV (95% CI)
Single outcomes		
Doctor diagnosis of asthma	82.1% (63.1% to 93.9%)	97.3% (92.4% to 99.4%)
Ever reported breathlessness	51.3% (34.8% to 67.6%)	94.1% (87.6% to 97.8%)
Ever reported wheezing	36.1% (25.1% to 48.3%)	100% (94.8% to 100%)
Wheezing reported on two or more occasions	46.0% (31.8% to 60.7%)	96.7% (90.7% to 99.3%)
Reported wheezing after age 3	47.2% (30.4% to 64.5%)	91.4% (84.4% to 96.0%)
Ever reported a frequent wheeze	50.0% (33.4% to 66.6%)	93.2% (86.5% to 97.2%)
Reported a frequent wheeze after age 3	75.0% (50.9% to 91.3%)	90.9% (84.3% to 95.4%)
Combinations of outcomes		
Ever wheezed+ever breathless	55.6% (38.1% to 72.1%)	94.3% (88.0% to 97.9%)
Ever symptoms+doctor diagnosis	90.0% (68.3% to 98.8%)	93.4% (87.4% to 97.1%)
Wheezing on ≥2 occasions+doctor diagnosis	87.0% (89.3% to 98.1%)	94.9% (89.3% to 98.1%)
Ever reported a frequent wheeze+doctor diagnosis	94.4% (72.7% to 99.9%)	92.7% (86.6% to 96.6%)

ALSPAC, Avon Longitudinal Study of Parents and Children; NPV, negative predictive value; PPV, positive predictive value.

Combining symptoms with reports of a doctor's diagnosis of asthma gave slightly higher specificities than the latter alone (table 4), but the sensitivity of each combination was somewhat lower. The combination with the highest Youden's index (sensitivity+specificity−100%) was wheezing reported on at least two occasions plus reports of a doctor's diagnosis (Youden's index 74.3%, compared with a figure of 84.2% for doctor's diagnosis alone). This combination also gave high predictive values, as did combining frequent wheeze with a doctor's diagnosis.

**Table 4:** Combination of outcomes versus GPRD asthma

ALSPAC asthma outcome	GP-recorded diagnosis of asthma	
	No	Yes
Ever reported wheezing+ever reported breathlessness		
No	99 (86.1%)	6
Yes	16	20 (76.9%)
Ever reported symptoms+doctor diagnosis by 7.5 years		
No	113 (98.3%)	8
Yes	2	18 (69.2%)
Wheezing reported on ≥2 occasions+doctor diagnosis		
No	112 (97.4%)	6
Yes	3	20 (76.9%)
Ever reported a frequent wheeze+doctor diagnosis		
No	114 (99.1%)	9
Yes	1	17 (65.4%)

ALSPAC, Avon Longitudinal Study of Parents and Children; GP, general practitioner; GPRD, General Practice Research Database.

Changing the age cut-off in GPRD from 9 to 8 years for the question ‘Has a doctor ever diagnosed your child with asthma?’ had only a small impact on the results: the sensitivity of this measure increased to 91.3% and the specificity decreased to 94.1%.

## Discussion

We have found that parental reports of a doctor's diagnosis accurately predicted the cumulative incidence of asthma in children with complete data recorded on asthma symptoms from birth to (8.5) years. Definitions consisting of wheeze or frequent wheeze reported on several occasions were more specific but had low sensitivity. This is unsurprising, as these criteria would favour more severe cases. Conversely, the sensitivity of ever wheezing and wheezing reported on two or more occasions was high but both had comparatively low specificities.

An issue in asthma validation studies is that there is no true gold standard. However, clinical assessment is regarded as the optimal standard.^3^ In this study, we have used linkage to the GPRD to identify ALSPAC participants with an asthma diagnosis in their GP record. Although diagnoses in the GPRD have been shown to be well recorded, particularly for chronic conditions,^15^ there are some weaknesses with this approach. Low population coverage of the GPRD meant that only a small proportion of ALSPAC participants were captured through this linkage. Further, linked individuals only had a complete record while registered with a practice contributing to the GPRD. Having said this, of 165 participants in this study who ever had an asthma diagnosis, 65 (39%) were dated before their GPRD registration date. Also, high agreement between parent reports and GP records suggest that missing historic diagnoses were not a substantial issue. A further shortcoming is the extent of missing ALSPAC data: complete data were only available for 29% of the linked participants with known asthma status. If those without complete data were less likely to complete the questionnaires accurately than those with complete data, then the levels of agreement presented here could be overestimates. However, the relative performance of the measures is unlikely to have been affected.

The main difference between this study and the majority of other asthma validation studies is that the symptom data in the latter were cross-sectional, so wheezing questions generally referred to the past 12 months and coincided with the timing of clinical assessment. These studies have generally concluded that wheezing questions are good at identifying asthmatics. A few studies have looked at ‘ever wheezing’ to estimate the cumulative incidence of asthma. In these studies, the sensitivity and specificity of ‘ever wheezing’ ranged from 80.6% to 94.5% and 74.9% to 89.9%, respectively.^5^ ^16–18^ However, in each case, this was based on a question asked at only one point in time. Jenkins *et al*^7^ found that reported wheezing was better at identifying adults than children with asthma and suggested that parents unfamiliar with wheezing may misreport other respiratory sounds as wheezing. Asking the question, ‘Has your child ever wheezed?’ at one time point is more likely to include children with persistent wheezing or asthma diagnosis due to difficulties of parental recall of sporadic wheeze in early childhood.^19^ Because we asked questions about wheezing throughout early childhood, this may have included a higher proportion of participants who had viral infections causing wheezing. It has been estimated that 30–50% of preschool children experience episodic viral wheeze at least once.^20^ This is supported by a Swedish study, where the prevalence of wheezing ranged from 26.6% among those aged 1–2 to 13.2% among those aged 5–6, whereas the prevalence of doctor-diagnosed asthma at these ages was 3.6% and 5.7%, respectively.^17^ Further, results from another study showed that 48.5% of children aged 6 years had ever wheezed but 41% of these had transient early wheezing—at least one lower respiratory tract infection with wheezing before the age of 3 but no wheezing at 6 years; the majority of this group had no increased risk of asthma in later life.^21^ Our results regarding reported doctor-diagnosed asthma generally agree with previously published results. One study reported a sensitivity of 76.9% and a specificity of 97.5% for current doctor-diagnosed asthma, as determined from medical records.^17^ Two recent studies of the cumulative incidence of childhood asthma determined from GP (UK-based) or health claims data (Canada) compared with parent-reported diagnosis reported high specificities but relatively low sensitivities.^22^ ^23^ Finally, de Marco *et al*^8^ found that the question ‘Have you ever had asthma?’ agreed most closely with clinical diagnosis.

Owing to the lack of firm diagnostic criteria, the measurement of asthma in epidemiological studies is problematic. Using ever wheezing measured longitudinally from early childhood as a proxy for asthma is likely to greatly overestimate its prevalence, whereas reports of a doctor's diagnosis will estimate it reasonably accurately. Our results suggest that there is no ‘ideal’ way to define asthma in terms of wheezing alone. A measure which correctly identifies the majority of non-asthmatics will not pick up a very large proportion of children with asthma, and vice versa. Having said this, the purpose of an epidemiological study is often to identify risk factors rather than to estimate prevalence. In general, relative risks will be biased towards the null in the presence of non-differential misclassification, and this is particularly dependent on a measure's specificity.^24^ Pekkanen and Pearce^3^ suggest that a highly specific measure could be achieved by using a combination of BHR and symptoms or BHR, symptoms and reported doctor diagnosis. In our study, combining symptoms or BHR with a doctor's diagnosis certainly achieved high specificities. However, these combinations were not very sensitive and a doctor's diagnosis performed almost as well in terms of specificity but misclassified fewer asthmatics. Further, very high specificities were achieved by combining wheezing outcomes with reports of a doctor's diagnosis, and these also misclassified fewer asthmatics. Peat *et al* suggest that using reports of a doctor's diagnosis is not a good way to detect participants with current severe asthma. They argue that identifying this group is important for determining risk factors and that this can be achieved with BHR.^25^ Our data do not support this, as 65% of those with BHR had never received a diagnosis of asthma. Similarly, only 74% of those with BHR had ever wheezed.

In conclusion, we have found that parental reports of a doctor's diagnosis of asthma agree well with clinical records; we recommend that this question is incorporated into epidemiological questionnaires to supplement symptom data. The use of wheezing to define asthma is more problematic but our results suggest that reasonably high specificity can be achieved by using conditional questions about wheezing, although at the cost of lower sensitivity and failure to identify (probably) milder cases. We have also shown that linking data from observational studies to electronic patient records can be an effective means of validating parent-reported data, as well as providing a source of outcome data that may otherwise be missing. However, it should be acknowledged that the success of this methodology relies on good coverage of relevant datasets as well as low levels of dissent from participants to link to these.

## Supplementary Material

Author's manuscript

Reviewer comments

## References

[1] British Thoracic Society Scottish Intercollegiate Guidelines Network. British guideline on the management of asthma. Thorax 2008;63 (Suppl 4):iv1–121 18463203 10.1136/thx.2008.097741

[2] Global Initiative for Asthma. Global strategy for asthma management and prevention 2011. http://www.ginasthma.org/uploads/users/files/GINA_Report_2011.pdf (accessed 5 Jun 2012)

[3] Pekkanen J Pearce N Defining asthma in epidemiological studies. Eur Respir J 1999;14:951–7 10573248 10.1034/j.1399-3003.1999.14d37.x

[4] Asher MI Keil U Anderson HR International Study of Asthma and Allergies in Childhood (ISAAC): rationale and methods. Eur Respir J 1995;8:483–91 7789502 10.1183/09031936.95.08030483

[5] Sole D Vanna AT Yamada E International Study of Asthma and Allergies in Childhood (ISAAC) written questionnaire: validation of the asthma component among Brazilian children. J Investig Allergol Clin Immunol 1998;8:376–82 10028486

[6] Remes ST Pekkanen J Remes K In search of childhood asthma: questionnaire, tests of bronchial hyperresponsiveness, and clinical evaluation. Thorax 2002;57:120–6 11828040 10.1136/thorax.57.2.120 PMC1746240

[7] Jenkins MA Clarke JR Carlin JB Validation of questionnaire and bronchial hyperresponsiveness against respiratory physician assessment in the diagnosis of asthma. Int J Epidemiol 1996;25:609–16 8671563 10.1093/ije/25.3.609

[8] de Marco R Cerveri I Bugiani M An undetected burden of asthma in Italy: the relationship between clinical and epidemiological diagnosis of asthma. Eur Respir J 1998;11:599–605 9596109

[9] Elphick HE Ritson S Rodgers H When a ‘wheeze’ is not a wheeze: acoustic analysis of breath sounds in infants. Eur Respir J 2000;16:593–7 11106197 10.1034/j.1399-3003.2000.16d04.x

[10] Lowe L Murray CS Martin L Reported versus confirmed wheeze and lung function in early life. Arch Dis Child 2004;89:540–3 15155399 10.1136/adc.2003.038539 PMC1719938

[11] Toelle BG Peat JK Salome CM Toward a definition of asthma for epidemiology. Am Rev Respir Dis 1992;146:633–7 1519839 10.1164/ajrccm/146.3.633

[12] Boyd A Golding J Macleod J Cohort profile: the ‘Children of the 90s’—the index offspring of the Avon Longitudinal Study of Parents and Children. Int J Epidemiol 2013;42:111–27 22507743 10.1093/ije/dys064 PMC3600618

[13] Yan K Salome C Woolcock AJ Rapid method for measurement of bronchial responsiveness. Thorax 1983;38:760–5 6648855 10.1136/thx.38.10.760 PMC459653

[14] Roberts G Peckitt C Northstone K Relationship between aeroallergen and food allergen sensitization in childhood. Clin Exp Allergy 2005;35:933–40 16008681 10.1111/j.1365-2222.2005.02280.x

[15] Khan NF Harrison SE Rose PW Validity of diagnostic coding within the General Practice Research Database: a systematic review. Br J Gen Pract 2010;60:e128–36 20202356 10.3399/bjgp10X483562 PMC2828861

[16] Mata Fernandez C Fernandez-Benitez M Perez Miranda M Validation of the Spanish version of the Phase III ISAAC questionnaire on asthma. J Investig Allergol Clin Immunol 2005;15:201–10 16261957

[17] Hederos CA Hasselgren M Hedlin G Comparison of clinically diagnosed asthma with parental assessment of children's asthma in a questionnaire. Pediatr Allergy Immunol 2007;18:135–41 17338786 10.1111/j.1399-3038.2006.00474.x

[18] Lukrafka JL Fuchs SC Moreira LB Performance of the ISAAC questionnaire to establish the prevalence of asthma in adolescents: a population-based study. J Asthma 2010;47:166–9 20170324 10.3109/02770900903483766

[19] Luyt DK Burton PR Simpson H Epidemiological study of wheeze, doctor diagnosed asthma, and cough in preschool children in Leicestershire. BMJ 1993;306:1386–90 8518607 10.1136/bmj.306.6889.1386 PMC1677841

[20] Townshend J Hails S McKean M Diagnosis of asthma in children. BMJ 2007;335:198–202 17656544 10.1136/bmj.39234.651412.AE PMC1934462

[21] Martinez FD Wright AL Taussig LM Asthma and wheezing in the first six years of life. N Engl J Med 1995;332:133–8 7800004 10.1056/NEJM199501193320301

[22] Yang CL To T Foty RG Verifying a questionnaire diagnosis of asthma in children using health claims data. BMC Pulm Med 2011;11:52 22108202 10.1186/1471-2466-11-52 PMC3233499

[23] Canova C Harris JM Mills P Epidemiological measures of childhood asthma: cross-sectional and longitudinal consistency. Respir Med 2012;106:1226–35 22705292 10.1016/j.rmed.2012.05.008

[24] Copeland KT Checkoway H McMichael AJ Bias due to misclassification in the estimation of relative risk. Am J Epidemiol 1977;105:488–95 871121 10.1093/oxfordjournals.aje.a112408

[25] Peat JK Toelle BG Marks GB Continuing the debate about measuring asthma in population studies. Thorax 2001;56:406–11 11312411 10.1136/thorax.56.5.406 PMC1746034

