# Supplementary material for: Validating childhood asthma in an epidemiological study using linked electronic patient records
Source: BMJ Open. 2014 Apr 23;4(4):e005345. doi: 10.1136/bmjopen-2014-005345 (PMC4010849; doi:10.1136/bmjopen-2014-005345)
Supplement: Author's manuscript [file bmjopen-2014-005345.draft_revisions.pdf]

BMJ Open

Validating childhood asthma in an epidemiological study using linked electronic patient records

Journal:	*BMJ Open*
Manuscript ID:	bmjopen-2014-005345
Article Type:	Research
Date Submitted by the Author:	26-Mar-2014
Complete List of Authors:	Cornish, Rosie; University of Bristol, School of Social and Community Medicine<br>Henderson, John; University of Bristol, School of Social and Community Medicine<br>Boyd, Andy; University of Bristol, School of Social and Community Medicine<br>Granell, Raquel; University of Bristol, School of Social and Community Medicine<br>Van Staa, Tjeerd; MHRA, CPRD<br>Macleod, John; University of Bristol, School of Social and Community Medicine
**Primary Subject Heading**:	Health informatics
Secondary Subject Heading:	Epidemiology, Respiratory medicine
Keywords:	EPIDEMIOLOGY, PRIMARY CARE, Asthma < THORACIC MEDICINE, Epidemiology < THORACIC MEDICINE

only

SCHOLARONE™
Manuscripts

Validating childhood asthma in an epidemiological study using linked electronic patient records

Rosaleen P Cornish[1], John Henderson[1], Andrew W Boyd[1], Raquel Granell[1], Tjeerd Van Staa[2], John Macleod[1*].

1. School of Social and Community Medicine, University of Bristol, UK
2. Clinical Practice Research Datalink (CPRD), Medicines and Healthcare Products Regulatory Agency, London, UK

*Corresponding author: Professor John Macleod, School of Social and Community Medicine, University of Bristol, Canynge Hall, 39 Whatley Road, Bristol, BS8 2PS, UK. Email: john.macleod@bristol.ac.uk Tel: +44(0)1173313935 Fax: +44(0)1173313914

Keywords: asthma, wheezing, record linkage, ALSPAC, GPRD

Word count: 3198

For peer review only

ABSTRACT

Objective

To investigate the performance of parent-reported data in identifying physician-confirmed asthma.

Design and setting

Validation study using linkage between the Avon Longitudinal Study of Parents and Children (ALSPAC) and electronic patient records held within the General Practice Research Database (GPRD).

Participants

Subjects were those eligible to participate in ALSPAC who also had a record in the GPRD; this included 765 individuals, just under 4% of ALSPAC-eligible subjects. The analysis was based on 141 subjects with complete parent-reported asthma data.

Primary and secondary outcome measures

The main GPRD outcome measure was the whether a child had a diagnosis of asthma before they were nine. Parent-reported measures were doctor diagnosis of asthma (before mean age 7.5 years), various outcomes based on wheezing and breathlessness recorded longitudinally between 6 months and 8.5 years. Secondary outcomes were bronchial hyper-responsiveness (BHR), FEV_1/FVC ratio and skin prick test responses.

Results

Among the 141 subjects with complete parent-reported data, 26 (18%) had an asthma diagnosis before age nine. Using GP-recorded asthma as the gold standard, the question "Has a doctor ever diagnosed your child with asthma?" was both sensitive (88.5%) and specific (95.7%). "Ever wheezed" had the highest sensitivity (100%) but low specificity (60.0%). More specific definitions were obtained by restricting to those who had wheezed on more than one occasion, experienced frequent wheeze, and/or wheezed after the age of 3, but these measures had low sensitivities. BHR only identified 50% of those with a GP-recorded diagnosis.

Conclusions

For peer review only - http://bmjopen.bmj.com/site/about/guidelines.xhtml

Parental reports of a doctor's diagnosis agree well with a GP-recorded diagnosis. High specificity for asthma can be achieved by using detailed wheezing questions, although these definitions are likely to exclude mild cases of asthma. Our study shows that linkage between observational studies and electronic patient records has the potential to enhance epidemiological research.

STRENGTHS AND LIMITATIONS OF THIS STUDY

- We were able to successfully link data from ALSPAC with the GPRD.
- The richness of the ALSPAC data allowed us to explore a number of different epidemiological constructs of asthma definition in relation to a recorded physician-diagnosis.
- We were able to compare the relative performance of objective measurements and lung function to that of parent-reported symptom data in identifying physician-confirmed asthma.
- Information on wheezing was collected longitudinally between ages 6 months and 8.5 years.
- The proportion of subjects with complete self-reported data from ALSPAC was relatively small.

INTRODUCTION

Asthma is difficult to measure in epidemiological studies because there are no definitive diagnostic criteria. The presence of recurrent respiratory symptoms, particularly wheezing and breathlessness, is central to its diagnosis in children. Bronchial hyper-responsiveness (BHR) is also a feature of asthma (1,2) but is not consistently present and can exist in the absence of clinical symptoms. Other measures of lung function may be normal during asymptomatic periods and can also be abnormal in the presence of other respiratory diseases.(1) Thus, due to the variable nature of asthma symptoms – both between and within individuals – its diagnosis remains a clinical one and clinical assessment is regarded as the best method for validating self-reported asthma data.(3)

For peer review only - http://bmjopen.bmj.com/site/about/guidelines.xhtml

However, clinical examination is costly and questionnaires are often preferred in epidemiological studies. Questions on wheezing, such as those used in the International Study of Asthma and Allergies in Childhood (ISAAC),(4) have been shown to provide valid measures of the prevalence of asthma in children.(5-8) Having said this, it is not possible to reliably distinguish between wheezing due to viral respiratory infections, which are common among young children, from wheezing due to asthma. In addition, mistaking other respiratory noises as wheezing can lead to over-reporting.(7,9,10) Consequently, it has been suggested that questionnaires should be supplemented by measurements of airway responsiveness.(11) However, several validation studies have shown that agreement between such measures and clinical asthma is poor.(3)

In the UK, childhood asthma is predominantly diagnosed and managed in primary care. Linkage to primary care records therefore provides a potential means to validate self-reported data. In this study we have compared longitudinal data on wheezing and other respiratory outcomes with linked electronic patient records to investigate the performance of epidemiological data in identifying physician-confirmed asthma.

METHODS

Subjects

Subjects were those eligible to participate in the Avon Longitudinal Study of Parents and Children (ALSPAC) who also had a record in the General Practice Research Database (GPRD) (now the Clinical Practice Research Datalink: http://www.cprd.com). The GPRD is an anonymised database of primary care records of around 5 million patients in the UK. ALSPAC has been described in detail before.(12) Briefly, just over 20,000 pregnant women living in and around Bristol, UK with due dates during 1991-1992 were eligible to take part; 15,247 pregnancies were recruited (15,390 fetuses, of which 14,701 were alive at 1 year) and these have been followed up since birth. (The ALSPAC website has a searchable data dictionary (http://www.bris.ac.uk/alspac/researchers/data-access/data-dictionary/

For peer review only - http://bmjopen.bmj.com/site/about/guidelines.xhtml

describing all available data.) Ethical approval was obtained from the ALSPAC Ethics and Law Committee, Local Research Ethics Committees and the NHS National Information Governance Board (NIGB) Ethics and Confidentiality Committee.

Linkage between ALSPAC and the GPRD

Linkage between ALSPAC and the GPRD was conducted by the NHS Information Centre (NHS IC) as a trusted third party, using a methodology to preserve anonymity. They had previously linked ALSPAC participants to the NHS Central Register, with a 99% match rate;(12) this was done on the basis of NHS ID number, name, date of birth and postcode using deterministic linkage. With approval from the NIGB Ethics and Confidentiality Committee, the NHS IC used this information to identify ALSPAC eligible individuals who also appeared in the GPRD; they then sent an anonymised linking dataset to be stored securely at the GPRD. ALSPAC and GPRD data for linked individuals were merged and analysed in a safe setting at the GPRD offices. As GPRD is anonymous and collected on an opt-out basis, and anonymity was preserved using the safeguards described above, this piece of research does not require consent above and beyond the consent obtained for participation in ALSPAC. However, ALSPAC has recently been collecting consent from participants, who are now adults, for ongoing participation in the study as well as consent to extract information from health and other administrative records and any participants who withdrew from the study or did not agree to their health records being extracted were excluded from the linkage.

ALSPAC data

When the children were, on average, $7\frac{1}{2}$ years old mothers were asked (via a postal questionnaire) whether a doctor had ever diagnosed their child with asthma. Data on asthma symptoms (wheezing and breathlessness) were collected at 6 months and approximately every 12 months thereafter (at 18, 30, 42, 57, 69, 81, 91 and 103 months). As well as asking whether the child had wheezed, the mothers were asked whether their child had experienced “wheezing with whistling on the chest

For peer review only - http://bmjopen.bmj.com/site/about/guidelines.xhtml

when (s)he breathed" and, if so, on how many occasions. Symptom questions referred to the previous 12 months, apart from at 6 and 57 months, which referred to the past 6 and 15 months, respectively. A child was defined to have wheezed if the response to either question about wheezing was yes and not wheezed if the response to both was no. Frequent wheeze was defined as wheezing with whistling on the chest on at least three occasions during the past 12 months. During a clinic attended between 8 and 9 years, FEV_1 and FVC were measured by spirometry and bronchial responsiveness was measured using the rapid methacholine inhalation test;(13) the dose-response slope of FEV_1 per µmol methacholine was used to classify subjects into four categories: none, low, moderate, and high, based on tertiles of the dose-response slope; BHR was defined as being the highest tertile. Sensitization to house dust mite, cat, and mixed grass was assessed by skin prick tests with a positive response defined as a mean weal diameter of 2mm or greater; this definition identified over 90% of subjects sensitized to any one of a panel of up to 12 allergens.(14) Symptom data were converted to "ever wheezed", "ever reported frequent wheeze" and "ever had breathlessness" – all defined as positive if there was a positive response at any time point and negative if all responses were negative. Subjects were classified according to whether or not their FEV_1/FVC ratio was less than 85% and whether or not they had at least one positive skin prick test.

GPRD data

Patients were defined as having a GP diagnosis of asthma if they had a Read code (the clinical coding system used by GPs in the UK – http://www.connectingforhealth.nhs.uk/systemsandservices/data/uktc/readcodes/index_html) indicating an asthma diagnosis (any Read code starting with "H33") in their record. In order to coincide with the timing of ALSPAC measurements, subjects were classified according to whether they had a diagnosis before 9 years of age. Thus, subjects who had no asthma diagnosis but left a GPRD practice before 9 were excluded as it could not be determined whether they were

For peer review only - http://bmjopen.bmj.com/site/about/guidelines.xhtml

subsequently diagnosed with asthma. Conversely, those who were not in the GPRD from birth were not excluded because historic diagnoses appeared in patients' records.

Statistical methods

Sensitivity, specificity and predictive values for ALSPAC outcomes and combinations of outcomes were calculated using GP-recorded diagnosis as the gold standard. Exact confidence intervals were calculated based on binomial probabilities. These analyses were restricted to those with complete parent-reported data from ALSPAC. Because the question about a doctor's diagnosis of asthma was asked, on average, a year earlier than the last wheezing question as well as before lung function was measured, this outcome was also compared to GP-recorded asthma before age 8 years in order to evaluate the likely impact of this age gap on the estimated sensitivity and specificity. In order to try to restrict to current asthma, we looked at whether children with an early diagnosis had evidence of persistent asthma by school age or beyond. There was only one child in the complete-case analysis with an early diagnosis (aged 3) but no further evidence of asthma. However, since this child left the GPRD while aged 6, it could not be determined whether or not they had persistent asthma and they were left in the analysis. Analyses were carried out using Stata 12.0.

RESULTS

Of the live births linked by the NHS IC, 765 appeared in the GPRD (this constitutes 4% of those eligible to take part in ALSPAC); four individuals did not want their health records accessed and one registered into and left the GPRD on the same day. Of the remaining 760 individuals, 61 without an asthma diagnosis transferred out of the GPRD before the age of 9, leaving 699 subjects with known asthma status. Of these, 488 (70%) had enrolled in ALSPAC. 251 (51%) of these had information on whether a doctor had ever diagnosed asthma, 141 (29%) had complete parent-reported asthma data; slightly fewer had BHR, FEV_1, FVC and skin prick test data. Characteristics of all linked individuals with known asthma status and those included in the analysis are shown in Table 1. Among

For peer review only - http://bmjopen.bmj.com/site/about/guidelines.xhtml

the former, there were 115 children with a diagnosis of asthma before age 9, giving a cumulative incidence of 16%. This figure was slightly higher (18%) among those with complete ALSPAC data; these children were also less likely to live in deprived areas, as indicated by the index of multiple deprivation (IMD 2007) (χ^2=18.8, p=0.001).

Table 1: Characteristics of all linked subjects with known asthma status compared to those with complete ALSPAC parent-reported data

GPRD-recorded characteristics	Linked subjects who did not exit GPRD before age 9 (n=699)	Subjects with complete ALSPAC parent-reported data[1] (n=141)
Sex – Male	348 (50%)	70 (50%)
IMD[2] quintile - least deprived	68 (13%)	24 (24%)
2nd	155 (30%)	30 (30%)
3rd	120 (24%)	27 (27%)
4th	58 (11%)	8 (8%)
Most deprived	109 (21%)	11 (11%)
Asthma – cumulative incidence	115 (16%)	26 (18%)

1. The denominator for IMD is slightly lower (510 for all linked subjects with GPRD asthma status at age 9; 100 for those with complete ALSPAC parent-reported data) as this variable is not complete within GPRD.

Table 2 shows the proportions with and without a GP diagnosis correctly identified by different ALSPAC variables, as well as the overall percentage reporting each outcome. Wheezing was common: 51% of the children had ever wheezed and 27% reported frequent wheeze on at least one occasion.

Table 2: Cross-tabulation of key ALSPAC asthma variables and GPRD asthma

ALSPAC asthma outcome		GP-recorded diagnosis of asthma		
		No	Yes	Overall
Doctor diagnosis of asthma by $7\frac{1}{2}$ years	No	110 (95.7%)	3	113
	Yes	5	23 (88.5%)	28 (19.9%)
Ever reported breathlessness in past 12 months	No	96 (83.5%)	6	102
	Yes	19	20 (76.9%)	39 (27.7%)
Ever reported wheezing in past 12 months	No	69 (60.0%)	0	69
	Yes	46	26 (100%)	72 (51.1%)
Wheezing reported on at least two occasions	No	88 (76.5%)	3	91
	Yes	27	23 (88.5%)	50 (35.5%)

For peer review only - http://bmjopen.bmj.com/site/about/guidelines.xhtml

Wheezing reported after age 3 years	No<br>Yes	96 (83.5%)<br>19	9<br>17 (65.4%)	105<br>36 (25.5%)
Ever reported frequent wheeze	No<br>Yes	96 (83.5%)<br>19	7<br>19 (73.1%)	103<br>38 (27.0%)
Reported frequent wheeze after age 3 years	No<br>Yes	110 (95.7%)<br>5	11<br>15 (57.7%)	121<br>20 (14.2%)

Of the ALSPAC variables, 'ever wheezed' had the highest sensitivity (100%; 95% confidence interval 86.8-100%) but low specificity (60.0%; 50.4-69.0%). The question "Has a doctor ever diagnosed your child with asthma?" was both sensitive (88.5%; 69.8-97.6%) and specific (95.7%; 90.1-98.6%). By restricting to those who had wheezed on more than one occasion, experienced frequent wheeze, or wheezed after the age of 3, more specific asthma definitions were obtained (Table 2 and Supplementary Table 1). However, the consequent losses in sensitivity were substantial. Positive and negative predictive values are given in Table 4. These confirm the above results. BHR only identified 50% of those with a GP diagnosis; the specificity was also relatively low (75%). The sensitivities of FEV_1/FVC ratio and skin prick tests were also low (Supplementary Table 2).

Combining symptoms with reports of a doctor's diagnosis of asthma gave slightly higher specificities than the latter alone (Table 3) but the sensitivity of each combination was somewhat lower. The combination with the highest Youden's index (sensitivity + specificity – 100%) was wheezing reported on at least two occasions plus reports of a doctor's diagnosis (Youden's index 74.3%, compared with a figure of 84.2% for doctor's diagnosis alone). This combination also gave high predictive values, as did combining frequent wheeze with a doctor's diagnosis.

For peer review only

Table 3: Combination of outcomes vs. GPRD asthma

ALSPAC asthma outcome		GP-recorded diagnosis of asthma	
		No	Yes
Ever reported wheezing<br>+ ever reported breathlessness	No<br>Yes	99 (86.1%)<br>16	6<br>20 (76.9%)
Ever reported symptoms +<br>doctor diagnosis by $7\frac{1}{2}$ years	No<br>Yes	113 (98.3%)<br>2	8<br>18 (69.2%)

For peer review only - http://bmjopen.bmj.com/site/about/guidelines.xhtml

Wheezing reported on ≥2 occasions + doctor diagnosis	No<br>Yes	112 (97.4%)<br>3	6<br>20 (76.9%)
Ever reported frequent wheeze + doctor diagnosis	No<br>Yes	114 (99.1%)<br>1	9<br>17 (65.4%)

Table 4: Positive (PPV) and negative predictive values (NPV) of different asthma outcome measures

ALSPAC asthma outcome	PPV (95% CI)	NPV (95% CI)
Single outcomes:		
Doctor diagnosis of asthma	82.1% (63.1-93.9)	97.3% (92.4-99.4)
Ever reported breathlessness	51.3% (34.8-67.6)	94.1% (87.6-97.8)
Ever reported wheezing	36.1% (25.1-48.3)	100% (94.8-100)
Wheezing reported on two or more occasions	46.0% (31.8-60.7)	96.7% (90.7-99.3)
Reported wheezing after age 3	47.2% (30.4-64.5)	91.4% (84.4-96.0)
Ever reported frequent wheeze	50.0% (33.4-66.6)	93.2% (86.5-97.2)
Reported frequent wheeze after age 3	75.0% (50.9-91.3)	90.9% (84.3-95.4)
Combinations of outcomes:		
Ever wheezed + ever breathless	55.6% (38.1-72.1)	94.3% (88.0-97.9)
Ever symptoms + doctor diagnosis	90.0% (68.3-98.8)	93.4% (87.4-97.1)
Wheezing on ≥2 occasions + doctor diagnosis	87.0% (89.3-98.1)	94.9% (89.3-98.1)
Ever reported frequent wheeze + doctor diagnosis	94.4% (72.7-99.9)	92.7% (86.6-96.6)

Changing the age cut-off in GPRD from 9 to 8 years for the question "Has a doctor ever diagnosed your child with asthma?" had only a small impact on the results: the sensitivity of this measure increased to 91.3% and the specificity decreased to 94.1%.

DISCUSSION

We have found that parental reports of a doctor's diagnosis accurately predicted the cumulative incidence of asthma in children with complete data recorded on asthma symptoms from birth to (8.5) years. Definitions consisting of wheeze or frequent wheeze reported on several occasions were more specific but had low sensitivity. This is unsurprising, as these criteria would favour more severe cases. Conversely, the sensitivity of ever wheezing and wheezing reported on two or more occasions was high but both had comparatively low specificities.

An issue in asthma validation studies is that there is no true gold standard. However, clinical assessment is regarded as the optimal standard.(3) In this study we have used linkage to the GPRD to

For peer review only - http://bmjopen.bmj.com/site/about/guidelines.xhtml

identify ALSPAC subjects with an asthma diagnosis in their GP record. Although diagnoses in the GPRD have been shown to be well recorded, particularly for chronic conditions,(15) there are some weaknesses with this approach. Low population coverage of the GPRD meant that only a small proportion of ALSPAC participants were captured through this linkage. Further, linked individuals only had a complete record while registered with a practice contributing to the GPRD. Having said this, out of 165 subjects in this study who ever had an asthma diagnosis, 65 (39%) were dated before their GPRD registration date. Also, high agreement between parent-reports and GP records suggest that missing historic diagnoses were not a substantial issue. A further shortcoming is the extent of missing ALSPAC data: complete data were only available for 29% of the linked subjects with known asthma status. If those without complete data were less likely to complete the questionnaires accurately than those with complete data then the levels of agreement presented here could be overestimates. However, the relative performance of the measures is unlikely to have been affected.

The main difference between this study and the majority of other asthma validation studies is that the symptom data in the latter were cross-sectional, so wheezing questions generally referred to the past 12 months and coincided with the timing of clinical assessment. These studies have generally concluded that wheezing questions are good at identifying asthmatics. A few studies have looked at “ever wheezing” to estimate the cumulative incidence of asthma. In these studies, the sensitivity and specificity of “ever wheezing” ranged from 80.6% to 94.5% and 74.9% to 89.9%, respectively.(5,16,17,18) However, in each case, this was based on a question asked at only one point in time. Jenkins et al found that reported wheezing was better at identifying adults than children with asthma and suggested that parents unfamiliar with wheezing may misreport other respiratory sounds as wheezing.(7) Asking the question, “Has your child ever wheezed?” at one time point is more likely to include children with persistent wheezing or asthma diagnosis due to difficulties of parental recall of sporadic wheeze in early childhood.(19) Because we asked questions about wheezing throughout early childhood, this may have included a higher proportion of subjects who

For peer review only - http://bmjopen.bmj.com/site/about/guidelines.xhtml

had viral infections causing wheezing. It has been estimated that 30-50% of preschool children experience episodic viral wheeze at least once.(20) This is supported by a Swedish study, where the prevalence of wheezing ranged from 26.6% among those aged 1-2 to 13.2% among those aged 5-6, whereas the prevalence of doctor-diagnosed asthma at these ages was 3.6% and 5.7%, respectively.(17) Further, results from another study showed that 48.5% of children aged 6 years had ever wheezed but 41% of these had transient early wheezing – at least one lower respiratory tract infection with wheezing before the age of 3 but no wheezing at 6 years; the majority of this group had no increased risk of asthma in later life(21). Our results regarding reported doctor-diagnosed asthma generally agree with previously published results. One study reported a sensitivity of 76.9% and a specificity of 97.5% for current doctor-diagnosed asthma, as determined from medical records.(17) Two recent studies of the cumulative incidence of childhood asthma determined from GP (UK-based) or health claims data (Canada) compared with parent-reported diagnosis reported high specificities but relatively low sensitivities.(22,23) Finally, de Marco et al found that the question "Have you ever had asthma?" agreed most closely with clinical diagnosis.(8)

Because of the lack of firm diagnostic criteria, the measurement of asthma in epidemiological studies is problematic. Using ever wheezing measured longitudinally from early childhood as a proxy for asthma is likely to greatly overestimate its prevalence, whereas reports of a doctor's diagnosis will estimate it reasonably accurately. Our results suggest that there is not an "ideal" way to define asthma in terms of wheezing alone. A measure which correctly identifies the majority of non-asthmatics will not pick up a very large proportion of children with asthma, and vice-versa. Having said this, the purpose of an epidemiological study is often to identify risk factors rather than to estimate prevalence. In general, relative risks will be biased towards the null in the presence of non-differential misclassification, and this is particularly dependent on a measure's specificity.(24) Pekkanen and Pearce suggest that a highly specific measure could be achieved by using a combination of BHR and symptoms or BHR, symptoms and reported doctor diagnosis.(3) In our

For peer review only - http://bmjopen.bmj.com/site/about/guidelines.xhtml

study, combining symptoms or BHR with a doctor's diagnosis certainly achieved high specificities. However, these combinations were not very sensitive and a doctor's diagnosis performed almost as well in terms of specificity but misclassified fewer asthmatics. Further, very high specificities were achieved by combining wheezing outcomes with reports of a doctor's diagnosis and these also misclassified fewer asthmatics. Peat et al suggest that using reports of a doctor's diagnosis is not good way to detect subjects with current severe asthma. They argue that identifying this group is important for determining risk factors and that this can be achieved with BHR.(25) Our data do not support this, as 65% of those with BHR had never received a diagnosis of asthma. Similarly, only 74% of those with BHR had ever wheezed.

In conclusion, we have found that parental reports of a doctor's diagnosis of asthma agree well with clinical records; we recommend that this question is incorporated into epidemiological questionnaires to supplement symptom data. The use of wheezing to define asthma is more problematic but our results suggest that reasonably high specificity can be achieved by using conditional questions about wheezing, although at the cost of lower sensitivity and failure to identify (probably) milder cases. We have also shown that linking data from observational studies to electronic patient records can be an effective means of validating parent-reported data, as well as providing a source of outcome data that may otherwise be missing. However, it should be acknowledged that the success of this methodology relies on good coverage of relevant datasets as well as low levels of dissent from participants to link to these.

ACKNOWLEDGEMENTS

We are extremely grateful to all the families who took part in this study, the midwives for their help in recruiting them, and the whole ALSPAC team, which includes interviewers, computer and laboratory technicians, clerical workers, research scientists, volunteers, managers, receptionists and nurses.

For peer review only - http://bmjopen.bmj.com/site/about/guidelines.xhtml

For peer review only

AUTHORS' CONTRIBUTIONS

RC carried out the statistical analysis and drafted the manuscript. JH contributed to the design of the study, interpretation of data and drafting of the paper. AB established the linkage process and contributed to the drafting of the paper. TVS established the linkage and data management process and reviewed the manuscript. RG cleaned, processed and advised on the ALSPAC asthma data and reviewed the manuscript. JM conceived the study and is PI of the Project to Enhance ALSPAC through Record Linkage (PEARL). All authors read and approved the final manuscript.

COMPETING INTERESTS

None

FUNDING

This work was supported by the Wellcome Trust (WT086118/Z/08/Z). The UK Medical Research Council (MRC), the Wellcome Trust and the University of Bristol currently provide core funding for ALSPAC (WT092731). Data collection is funded from a wide range of sources.

DATA SHARING

The relevant ALSPAC study data and statistical code are available from the first author; linked GPRD data are available through the MHRA safe haven as described in the paper. Participants gave informed consent for data sharing. Full information on the ethical and governance stipulations around access to and sharing of ALSPAC data are available on the ALSPAC website

For peer review only

REFERENCES

1. British Guideline on the Management of Asthma. *Thorax* 2008;**63**Suppl4:iv1-121 doi:10.1136/thx.2008.097741.
2. Global Initiative for Asthma. Global Strategy for Asthma Management and Prevention 2011. Available from: http://www.ginasthma.org/uploads/users/files/GINA_Report_2011.pdf. (accessed 5 Jun 2012)
3. Pekkanen J, Pearce N. Defining asthma in epidemiological studies. *Eur Respir J* 1999; **14**(4):951-7.
4. Asher MI, Keil U, Anderson HR et al. International Study of Asthma and Allergies in Childhood (ISAAC): rationale and methods. *Eur Respir J* 1995;**8**(3):483-91 doi:10.1183/09031936.95.08030483.
5. Sole D, Vanna AT, Yamada E et al. International Study of Asthma and Allergies in Childhood (ISAAC) written questionnaire: validation of the asthma component among Brazilian children. *J Investig Allergol Clin Immunol* 1998;**8**(6):376-82.
6. Remes ST, Pekkanen J, Remes K et al. In search of childhood asthma: questionnaire, tests of bronchial hyperresponsiveness, and clinical evaluation. *Thorax* 2002;**57**(2):120-6 doi:10.1136/thorax.57.2.120.
7. Jenkins MA, Clarke JR, Carlin JB et al. Validation of questionnaire and bronchial hyperresponsiveness against respiratory physician assessment in the diagnosis of asthma. *Int J Epidemiol* 1996;**25**(3):609-16 doi:10.1093/ije/25.3.609.
8. de Marco R, Cerveri I, Bugiani M et al. An undetected burden of asthma in Italy: the relationship between clinical and epidemiological diagnosis of asthma. *Eur Respir J* 1998;**11**(3):599-605 doi:10.1183/09031936.98.11030599.
9. Elphick HE, Ritson S, Rodgers H et al. When a "wheeze" is not a wheeze: acoustic analysis of breath sounds in infants. *Eur Respir J* 2000;**16**(4):593-7.

For peer review only - http://bmjopen.bmj.com/site/about/guidelines.xhtml

For peer review only

10. Lowe L, Murray CS, Martin L et al. Reported versus confirmed wheeze and lung function in early life. *Arch Dis Child* 2004;**89**(6):540-3 doi:10.1136/adc.2003.038539.

11. Toelle BG, Peat JK, Salome CM et al. Toward a definition of asthma for epidemiology. *Am Rev Respir Dis* 1992;**146**(3):633-7 doi:10.1164/ajrccm/146.3.633.

12. Boyd A, Golding J, Macleod J et al. Cohort Profile: The 'Children of the 90s'—the index offspring of the Avon Longitudinal Study of Parents and Children. *Int J Epidemiol* 2013;**42**(1): 111-127 doi:10.1093/ije/dys064 [published Online First: April 16, 2012].

13. Yan K, Salome C, Woolcock AJ. Rapid method for measurement of bronchial responsiveness. *Thorax* 1983;**38**(10):760-5 doi:10.1136/thx.38.10.760

14. Roberts G, Peckitt C, Northstone K et al. Relationship between aeroallergen and food allergen sensitization in childhood. *Clin Exp Allergy* 2005;**35**(7):933-940 doi:10.1111/j.1365-2222.2005.02280.x [published Online First: July 6th 2005].

15. Khan NF, Harrison SE, Rose PW. Validity of diagnostic coding within the General Practice Research Database: a systematic review. *Br J Gen Pract* 2010;**60**(572):e128-36 doi:10.3399/bjgp10X483562.

16. Mata Fernandez C, Fernandez-Benitez M, Perez Miranda M et al. Validation of the Spanish version of the Phase III ISAAC questionnaire on asthma. *J Investig Allergol Clin Immunol* 2005;**15**(3):201-10.

17. Hederos CA, Hasselgren M, Hedlin G et al. Comparison of clinically diagnosed asthma with parental assessment of children's asthma in a questionnaire. *Pediatr Allergy Immunol* 2007;**18**(2):135-41 doi: 10.1111/j.1399-3038.2006.00474.x [published Online First: 26 Oct 2006].

18. Lukrafka JL, Fuchs SC, Moreira LB et al. Performance of the ISAAC questionnaire to establish the prevalence of asthma in adolescents: a population-based study. *J Asthma* 2010;**47**(2):166-9 doi:10.3109/02770900903483766.

For peer review only - http://bmjopen.bmj.com/site/about/guidelines.xhtml

19. Luyt DK, Burton PR, Simpson H. Epidemiological study of wheeze, doctor diagnosed asthma, and cough in preschool children in Leicestershire. *BMJ* 1993;**306**(6889):1386-90 10.1136/bmj.306.6889.1386.

20. Townshend J, Hails S, McKean M. Diagnosis of asthma in children. *BMJ* 2007;**335**(7612):198-202 doi:10.1136/bmj.39234.651412.AE.

21. Martinez FD, Wright AL, Taussig LM et al. Asthma and Wheezing in the First Six Years of Life. *N Engl J Med* 1995;**332**(3):133-8 doi:10.1056/NEJM199501193320301.

22. Yang CL, To T, Foty RG et al. Verifying a questionnaire diagnosis of asthma in children using health claims data. *BMC Pulm Med* 2011;11:52 doi:10.1186/1471-2466-11-52.

23. Canova C, Harris JM, Mills P et al. Epidemiological measures of childhood asthma: Cross-sectional and longitudinal consistency. *Respir Med* 2012;**106**(9):1226-1235 doi:10.1016/j.rmed.2012.05.008 [published Online First: 16 June 2012].

24. Copeland KT, Checkoway H, McMichael AJ et al. Bias due to misclassification in the estimation of relative risk. *Am J Epidemiol* 1977;**105**(5):488-95.

25. Peat JK, Toelle BG, Marks GB et al. Continuing the debate about measuring asthma in population studies. *Thorax* 2001;**56**(5):406-11 doi:10.1136/thorax.56.5.406.

Licence statement

The Corresponding Author has the right to grant on behalf of all authors and does grant on behalf of all authors, an exclusive licence (or non-exclusive for government employees) on a worldwide basis to the BMJ Group and co-owners or contracting owning societies (where published by the BMJ Group on their behalf), and its Licensees to permit this article (if accepted) to be published in any BMJ Group products and to exploit all subsidiary rights, as set out in our licence.

For peer review only - http://bmjopen.bmj.com/site/about/guidelines.xhtml

For peer review only

Supplementary tables

Supplementary Table 1: Cross-tabulation of additional wheezing outcomes and GPRD asthma

ALSPAC asthma outcome		GP-recorded diagnosis of asthma		
		No	Yes	Overall
Wheezing reported on at least three occasions	No	101 (87.8%)	8	109
	Yes	14	18 (69.2%)	32 (22.7%)
Wheezing reported on at least four occasions	No	106 (92.1%)	15	121
	Yes	9	11 (42.3%)	20 (14.2%)
Wheezing reported after age 3 on at least two occasions	No	107 (93.0%)	11	118
	Yes	8	15 (57.7%)	23 (16.3%)
Reported frequent wheeze on at least two occasions	No	108 (93.9%)	12	130
	Yes	7	14 (53.8%)	21 (14.9%)
Reported frequent wheeze on at least three occasions	No	115 (100%)	19	134
	Yes	0	7 (26.9%)	7 (5.0%)
Reported frequent wheeze after age 3 years on at least two occasions	No	113 (98.3%)	16	129
	Yes	2	10 (38.5%)	12 (8.5%)

Supplementary Table 2: Cross-tabulation of objective measurements (and BHR combined with parent report of a doctor's diagnosis) and GPRD asthma

ALSPAC asthma outcome		GP-recorded diagnosis of asthma		
		No	Yes	Overall
BHR	No	44 (74.6%)	8	52
	Yes	15	8 (50.0%)	23 (30.7%)
FEV_1:FVC ratio < 85%	No	66 (78.6%)	15	81
	Yes	18	8 (34.8%)	26 (23.9%)
Any positive skin prick test	No	68 (82.9%)	13	81
	Yes	14	4 (23.5%)	18 (18.2%)
Doctor diagnosis by $7\frac{1}{2}$ years + BHR	No	110 (99.1%)	10	120
	Yes	1	8 (44.4%)	9 (7.0%)

For peer review only - http://bmjopen.bmj.com/site/about/guidelines.xhtml

STARD checklist for reporting of studies of diagnostic accuracy
(version January 2003)

Section and Topic	Item #		On page #
TITLE/ABSTRACT/ KEYWORDS	1	Identify the article as a study of diagnostic accuracy (recommend MeSH heading 'sensitivity and specificity').	Title page – indicates it is a validation study
INTRODUCTION	2	State the research questions or study aims, such as estimating diagnostic accuracy or comparing accuracy between tests or across participant groups.	4
METHODS			
Participants	3	The study population: The inclusion and exclusion criteria, setting and locations where data were collected.	4 & 6
	4	Participant recruitment: Was recruitment based on presenting symptoms, results from previous tests, or the fact that the participants had received the index tests or the reference standard?	4
	5	Participant sampling: Was the study population a consecutive series of participants defined by the selection criteria in item 3 and 4? If not, specify how participants were further selected.	4
	6	Data collection: Was data collection planned before the index test and reference standard were performed (prospective study) or after (retrospective study)?	4-6
Test methods	7	The reference standard and its rationale.	4 & 6
	8	Technical specifications of material and methods involved including how and when measurements were taken, and/or cite references for index tests and reference standard.	4-6
	9	Definition of and rationale for the units, cut-offs and/or categories of the results of the index tests and the reference standard.	4-6
	10	The number, training and expertise of the persons executing and reading the index tests and the reference standard.	5-6: Index "test" = self-report 6: Reference = GP data
	11	Whether or not the readers of the index tests and reference standard were blind (masked) to the results of the other test and describe any other clinical information available to the readers.	N/A – blinded by nature of study design
Statistical methods	12	Methods for calculating or comparing measures of diagnostic accuracy, and the statistical methods used to quantify uncertainty (e.g. 95% confidence intervals).	7
	13	Methods for calculating test reproducibility, if done.	N/A
RESULTS			
Participants	14	When study was performed, including beginning and end dates of recruitment.	4-6
	15	Clinical and demographic characteristics of the study population (at least information on age, gender, spectrum of presenting symptoms).	7-8
	16	The number of participants satisfying the criteria for inclusion who did or did not undergo the index tests and/or the reference standard; describe why participants failed to undergo either test (a flow diagram is strongly recommended).	7
Test results	17	Time-interval between the index tests and the reference standard, and any treatment administered in between.	N/A
	18	Distribution of severity of disease (define criteria) in those with the target condition; other diagnoses in participants without the target condition.	Not determined
	19	A cross tabulation of the results of the index tests (including indeterminate and missing results) by the results of the reference standard; for continuous results, the distribution of the test results by the results of the reference standard.	8-9
	20	Any adverse events from performing the index tests or the reference standard.	N/A

For peer review only

Estimates	21	Estimates of diagnostic accuracy and measures of statistical uncertainty (e.g. 95% confidence intervals).	9
	22	How indeterminate results, missing data and outliers of the index tests were handled.	6-7
	23	Estimates of variability of diagnostic accuracy between subgroups of participants, readers or centers, if done.	N/A
	24	Estimates of test reproducibility, if done.	N/A
DISCUSSION	25	Discuss the clinical applicability of the study findings.	N/A – study has epidemiological rather than clinical implications

For peer review only

Validating childhood asthma in an epidemiological study using linked electronic patient records

Rosaleen P Cornish[1], John Henderson[1], Andrew W Boyd[1], Raquel Granell[1], Tjeerd Van Staa[2], John Macleod[1*].

1. School of Social and Community Medicine, University of Bristol, UK
2. Clinical Practice Research Datalink (CPRD), Medicines and Healthcare Products Regulatory Agency, London, UK

*Corresponding author: Professor John Macleod, School of Social and Community Medicine, University of Bristol, Canynge Hall, 39 Whatley Road, Bristol, BS8 2PS, UK. Email: john.macleod@bristol.ac.uk Tel: +44(0)1173313935 Fax: +44(0)1173313914

Keywords: asthma, wheezing, record linkage, ALSPAC, GPRD

Word count: 3198

For peer review only

ABSTRACT

Objective

To investigate the performance of parent-reported data in identifying physician-confirmed asthma.

Design and setting

Validation study using linkage between the Avon Longitudinal Study of Parents and Children (ALSPAC) and electronic patient records held within the General Practice Research Database (GPRD).

Participants

Subjects were those eligible to participate in ALSPAC who also had a record in the GPRD; this included 765 individuals, just under 4% of ALSPAC-eligible subjects. The analysis was based on 141 subjects with complete parent-reported asthma data.

Primary and secondary outcome measures

The main GPRD outcome measure was the whether a child had a diagnosis of asthma before they were nine. Parent-reported measures were doctor diagnosis of asthma (before mean age 7.5 years), various outcomes based on wheezing and breathlessness recorded longitudinally between 6 months and 8.5 years. Secondary outcomes were bronchial hyper-responsiveness (BHR), FEV_1/FVC ratio and skin prick test responses.

Results

Among the 141 subjects with complete parent-reported data, 26 (18%) had an asthma diagnosis before age nine. Using GP-recorded asthma as the gold standard, the question "Has a doctor ever diagnosed your child with asthma?" was both sensitive (88.5%) and specific (95.7%). "Ever wheezed" had the highest sensitivity (100%) but low specificity (60.0%). More specific definitions were obtained by restricting to those who had wheezed on more than one occasion, experienced frequent wheeze, and/or wheezed after the age of 3, but these measures had low sensitivities. BHR only identified 50% of those with a GP-recorded diagnosis.

Conclusions

For peer review only - http://bmjopen.bmj.com/site/about/guidelines.xhtml

Parental reports of a doctor's diagnosis agree well with a GP-recorded diagnosis. High specificity for asthma can be achieved by using detailed wheezing questions, although these definitions are likely to exclude mild cases of asthma. Our study shows that linkage between observational studies and electronic patient records has the potential to enhance epidemiological research.

STRENGTHS AND LIMITATIONS OF THIS STUDY

- We were able to successfully link data from ALSPAC with the GPRD.
- The richness of the ALSPAC data allowed us to explore a number of different epidemiological constructs of asthma definition in relation to a recorded physician-diagnosis.
- We were able to compare the relative performance of objective measurements and lung function to that of parent-reported symptom data in identifying physician-confirmed asthma.
- Information on wheezing was collected longitudinally between ages 6 months and 8.5 years.
- The proportion of subjects with complete self-reported data from ALSPAC was relatively small.

INTRODUCTION

Asthma is difficult to measure in epidemiological studies because there are no definitive diagnostic criteria. The presence of recurrent respiratory symptoms, particularly wheezing and breathlessness, is central to its diagnosis in children. Bronchial hyper-responsiveness (BHR) is also a feature of asthma (1,2) but is not consistently present and can exist in the absence of clinical symptoms. Other measures of lung function may be normal during asymptomatic periods and can also be abnormal in the presence of other respiratory diseases.(1) Thus, due to the variable nature of asthma symptoms – both between and within individuals – its diagnosis remains a clinical one and clinical assessment is regarded as the best method for validating self-reported asthma data.(1)

For peer review only - http://bmjopen.bmj.com/site/about/guidelines.xhtml

However, clinical examination is costly and questionnaires are often preferred in epidemiological studies. Questions on wheezing, such as those used in the International Study of Asthma and Allergies in Childhood (ISAAC),(4) have been shown to provide valid measures of the prevalence of asthma in children.(5-8) Having said this, it is not possible to reliably distinguish between wheezing due to viral respiratory infections, which are common among young children, from wheezing due to asthma. In addition, mistaking other respiratory noises as wheezing can lead to over-reporting.(7,9,10) Consequently, it has been suggested that questionnaires should be supplemented by measurements of airway responsiveness.(11) However, several validation studies have shown that agreement between such measures and clinical asthma is poor.(3)

In the UK, childhood asthma is predominantly diagnosed and managed in primary care. Linkage to primary care records therefore provides a potential means to validate self-reported data. In this study we have compared longitudinal data on wheezing and other respiratory outcomes with linked electronic patient records to investigate the performance of epidemiological data in identifying physician-confirmed asthma.

METHODS

Subjects

Subjects were those eligible to participate in the Avon Longitudinal Study of Parents and Children (ALSPAC) who also had a record in the General Practice Research Database (GPRD) (now the Clinical Practice Research Datalink: http://www.cprd.com). The GPRD is an anonymised database of primary care records of around 5 million patients in the UK. ALSPAC has been described in detail before.(12) Briefly, just over 20,000 pregnant women living in and around Bristol, UK with due dates during 1991-1992 were eligible to take part; 15,247 pregnancies were recruited (15,390 fetuses, of which 14,701 were alive at 1 year) and these have been followed up since birth. (The ALSPAC website has a searchable data dictionary (http://www.bris.ac.uk/alspac/researchers/data-access/data-dictionary/

For peer review only - http://bmjopen.bmj.com/site/about/guidelines.xhtml

describing all available data.) Ethical approval was obtained from the ALSPAC Ethics and Law Committee, Local Research Ethics Committees and the NHS National Information Governance Board (NIGB) Ethics and Confidentiality Committee.

Linkage between ALSPAC and the GPRD

Linkage between ALSPAC and the GPRD was conducted by the NHS Information Centre (NHS IC) as a trusted third party, using a methodology to preserve anonymity. They had previously linked ALSPAC participants to the NHS Central Register, with a 99% match rate;(12) this was done on the basis of NHS ID number, name, date of birth and postcode using deterministic linkage. With approval from the NIGB Ethics and Confidentiality Committee, the NHS IC used this information to identify ALSPAC eligible individuals who also appeared in the GPRD; they then sent an anonymised linking dataset to be stored securely at the GPRD. ALSPAC and GPRD data for linked individuals were merged and analysed in a safe setting at the GPRD offices. As GPRD is anonymous and collected on an opt-out basis, and anonymity was preserved using the safeguards described above, this piece of research does not require consent above and beyond the consent obtained for participation in ALSPAC. However, ALSPAC has recently been collecting consent from participants, who are now adults, for ongoing participation in the study as well as consent to extract information from health and other administrative records and any participants who withdrew from the study or did not agree to their health records being extracted were excluded from the linkage.

ALSPAC data

When the children were, on average, $7\frac{1}{2}$ years old mothers were asked (via a postal questionnaire) whether a doctor had ever diagnosed their child with asthma. Data on asthma symptoms (wheezing and breathlessness) were collected at 6 months and approximately every 12 months thereafter (at 18, 30, 42, 57, 69, 81, 91 and 103 months). As well as asking whether the child had wheezed, the mothers were asked whether their child had experienced “wheezing with whistling on the chest

For peer review only - http://bmjopen.bmj.com/site/about/guidelines.xhtml

when (s)he breathed" and, if so, on how many occasions. Symptom questions referred to the previous 12 months, apart from at 6 and 57 months, which referred to the past 6 and 15 months, respectively. A child was defined to have wheezed if the response to either question about wheezing was yes and not wheezed if the response to both was no. Frequent wheeze was defined as wheezing with whistling on the chest on at least three occasions during the past 12 months. During a clinic attended between 8 and 9 years, FEV_1 and FVC were measured by spirometry and bronchial responsiveness was measured using the rapid methacholine inhalation test;(13) the dose-response slope of FEV_1 per µmol methacholine was used to classify subjects into four categories: none, low, moderate, and high, based on tertiles of the dose-response slope; BHR was defined as being the highest tertile. Sensitization to house dust mite, cat, and mixed grass was assessed by skin prick tests with a positive response defined as a mean weal diameter of 2mm or greater; this definition identified over 90% of subjects sensitized to any one of a panel of up to 12 allergens.(14) Symptom data were converted to "ever wheezed", "ever reported frequent wheeze" and "ever had breathlessness" – all defined as positive if there was a positive response at any time point and negative if all responses were negative. Subjects were classified according to whether or not their FEV_1/FVC ratio was less than 85% and whether or not they had at least one positive skin prick test.

GPRD data

Patients were defined as having a GP diagnosis of asthma if they had a Read code (the clinical coding system used by GPs in the UK – http://www.connectingforhealth.nhs.uk/systemsandservices/data/uktc/readcodes/index_html) indicating an asthma diagnosis (any Read code starting with "H33") in their record. In order to coincide with the timing of ALSPAC measurements, subjects were classified according to whether they had a diagnosis before 9 years of age. Thus, subjects who had no asthma diagnosis but left a GPRD practice before 9 were excluded as it could not be determined whether they were

For peer review only - http://bmjopen.bmj.com/site/about/guidelines.xhtml

subsequently diagnosed with asthma. Conversely, those who were not in the GPRD from birth were not excluded because historic diagnoses appeared in patients' records.

Statistical methods

Sensitivity, specificity and predictive values for ALSPAC outcomes and combinations of outcomes were calculated using GP-recorded diagnosis as the gold standard. Exact confidence intervals were calculated based on binomial probabilities. These analyses were restricted to those with complete parent-reported data from ALSPAC. Because the question about a doctor's diagnosis of asthma was asked, on average, a year earlier than the last wheezing question as well as before lung function was measured, this outcome was also compared to GP-recorded asthma before age 8 years in order to evaluate the likely impact of this age gap on the estimated sensitivity and specificity. In order to try to restrict to current asthma, we looked at whether children with an early diagnosis had evidence of persistent asthma by school age or beyond. There was only one child in the complete-case analysis with an early diagnosis (aged 3) but no further evidence of asthma. However, since this child left the GPRD while aged 6, it could not be determined whether or not they had persistent asthma and they were left in the analysis. Analyses were carried out using Stata 12.0.

RESULTS

Of the live births linked by the NHS IC, 765 appeared in the GPRD (this constitutes 4% of those eligible to take part in ALSPAC); four individuals did not want their health records accessed and one registered into and left the GPRD on the same day. Of the remaining 760 individuals, 61 without an asthma diagnosis transferred out of the GPRD before the age of 9, leaving 699 subjects with known asthma status. Of these, 488 (70%) had enrolled in ALSPAC. 251 (51%) of these had information on whether a doctor had ever diagnosed asthma, 141 (29%) had complete parent-reported asthma data; slightly fewer had BHR, FEV_1, FVC and skin prick test data. Characteristics of all linked individuals with known asthma status and those included in the analysis are shown in Table 1. Among

For peer review only - http://bmjopen.bmj.com/site/about/guidelines.xhtml

the former, there were 115 children with a diagnosis of asthma before age 9, giving a cumulative incidence of 16%. This figure was slightly higher (18%) among those with complete ALSPAC data; these children were also less likely to live in deprived areas, as indicated by the index of multiple deprivation (IMD 2007) (χ^2=18.8, p=0.001).

Table 1: Characteristics of all linked subjects with known asthma status compared to those with complete ALSPAC parent-reported data

GPRD-recorded characteristics	Linked subjects who did not exit GPRD before age 9 (n=699)	Subjects with complete ALSPAC parent-reported data[1] (n=141)
Sex – Male	348 (50%)	70 (50%)
IMD[2] quintile - least deprived	68 (13%)	24 (24%)
2nd	155 (30%)	30 (30%)
3rd	120 (24%)	27 (27%)
4th	58 (11%)	8 (8%)
Most deprived	109 (21%)	11 (11%)
Asthma – cumulative incidence	115 (16%)	26 (18%)

1. The denominator for IMD is slightly lower (510 for all linked subjects with GPRD asthma status at age 9; 100 for those with complete ALSPAC parent-reported data) as this variable is not complete within GPRD.

Table 2 shows the proportions with and without a GP diagnosis correctly identified by different ALSPAC variables, as well as the overall percentage reporting each outcome. Wheezing was common: 51% of the children had ever wheezed and 27% reported frequent wheeze on at least one occasion.

Table 2: Cross-tabulation of key ALSPAC asthma variables and GPRD asthma

ALSPAC asthma outcome		GP-recorded diagnosis of asthma		
		No	Yes	Overall
Doctor diagnosis of asthma	No	110 (95.7%)	3	113
by $7\frac{1}{2}$ years	Yes	5	23 (88.5%)	28 (19.9%)
Ever reported breathlessness	No	96 (83.5%)	6	102
in past 12 months	Yes	19	20 (76.9%)	39 (27.7%)
Ever reported wheezing	No	69 (60.0%)	0	69
in past 12 months	Yes	46	26 (100%)	72 (51.1%)
Wheezing reported on at least two	No	88 (76.5%)	3	91
occasions	Yes	27	23 (88.5%)	50 (35.5%)

For peer review only - http://bmjopen.bmj.com/site/about/guidelines.xhtml

Wheezing reported after age 3 years	No<br>Yes	96 (83.5%)<br>19	9<br>17 (65.4%)	105<br>36 (25.5%)
Ever reported frequent wheeze	No<br>Yes	96 (83.5%)<br>19	7<br>19 (73.1%)	103<br>38 (27.0%)
Reported frequent wheeze after age 3 years	No<br>Yes	110 (95.7%)<br>5	11<br>15 (57.7%)	121<br>20 (14.2%)

Of the ALSPAC variables, 'ever wheezed' had the highest sensitivity (100%; 95% confidence interval 86.8-100%) but low specificity (60.0%; 50.4-69.0%). The question "Has a doctor ever diagnosed your child with asthma?" was both sensitive (88.5%; 69.8-97.6%) and specific (95.7%; 90.1-98.6%). By restricting to those who had wheezed on more than one occasion, experienced frequent wheeze, or wheezed after the age of 3, more specific asthma definitions were obtained (Table 2 and Supplementary Table 1). However, the consequent losses in sensitivity were substantial. Positive and negative predictive values are given in Table 4. These confirm the above results. BHR only identified 50% of those with a GP diagnosis; the specificity was also relatively low (75%). The sensitivities of FEV_1/FVC ratio and skin prick tests were also low (Supplementary Table 2).

Combining symptoms with reports of a doctor's diagnosis of asthma gave slightly higher specificities than the latter alone (Table 3) but the sensitivity of each combination was somewhat lower. The combination with the highest Youden's index (sensitivity + specificity – 100%) was wheezing reported on at least two occasions plus reports of a doctor's diagnosis (Youden's index 74.3%, compared with a figure of 84.2% for doctor's diagnosis alone). This combination also gave high predictive values, as did combining frequent wheeze with a doctor's diagnosis.

Table 3: Combination of outcomes vs. GPRD asthma

ALSPAC asthma outcome		GP-recorded diagnosis of asthma	
		No	Yes
Ever reported wheezing<br>+ ever reported breathlessness	No<br>Yes	99 (86.1%)<br>16	6<br>20 (76.9%)
Ever reported symptoms +<br>doctor diagnosis by $7\frac{1}{2}$ years	No<br>Yes	113 (98.3%)<br>2	8<br>18 (69.2%)

For peer review only - http://bmjopen.bmj.com/site/about/guidelines.xhtml

Wheezing reported on ≥2 occasions + doctor diagnosis	No	112 (97.4%)	6
	Yes	3	20 (76.9%)
Ever reported frequent wheeze + doctor diagnosis	No	114 (99.1%)	9
	Yes	1	17 (65.4%)

Table 4: Positive (PPV) and negative predictive values (NPV) of different asthma outcome measures

ALSPAC asthma outcome	PPV (95% CI)	NPV (95% CI)
Single outcomes:		
Doctor diagnosis of asthma	82.1% (63.1-93.9)	97.3% (92.4-99.4)
Ever reported breathlessness	51.3% (34.8-67.6)	94.1% (87.6-97.8)
Ever reported wheezing	36.1% (25.1-48.3)	100% (94.8-100)
Wheezing reported on two or more occasions	46.0% (31.8-60.7)	96.7% (90.7-99.3)
Reported wheezing after age 3	47.2% (30.4-64.5)	91.4% (84.4-96.0)
Ever reported frequent wheeze	50.0% (33.4-66.6)	93.2% (86.5-97.2)
Reported frequent wheeze after age 3	75.0% (50.9-91.3)	90.9% (84.3-95.4)
Combinations of outcomes:		
Ever wheezed + ever breathless	55.6% (38.1-72.1)	94.3% (88.0-97.9)
Ever symptoms + doctor diagnosis	90.0% (68.3-98.8)	93.4% (87.4-97.1)
Wheezing on ≥2 occasions + doctor diagnosis	87.0% (89.3-98.1)	94.9% (89.3-98.1)
Ever reported frequent wheeze + doctor diagnosis	94.4% (72.7-99.9)	92.7% (86.6-96.6)

Changing the age cut-off in GPRD from 9 to 8 years for the question "Has a doctor ever diagnosed your child with asthma?" had only a small impact on the results: the sensitivity of this measure increased to 91.3% and the specificity decreased to 94.1%.

DISCUSSION

We have found that parental reports of a doctor's diagnosis accurately predicted the cumulative incidence of asthma in children with complete data recorded on asthma symptoms from birth to (8.5) years. Definitions consisting of wheeze or frequent wheeze reported on several occasions were more specific but had low sensitivity. This is unsurprising, as these criteria would favour more severe cases. Conversely, the sensitivity of ever wheezing and wheezing reported on two or more occasions was high but both had comparatively low specificities.

An issue in asthma validation studies is that there is no true gold standard. However, clinical assessment is regarded as the optimal standard.(3) In this study we have used linkage to the GPRD to

For peer review only - http://bmjopen.bmj.com/site/about/guidelines.xhtml

identify ALSPAC subjects with an asthma diagnosis in their GP record. Although diagnoses in the GPRD have been shown to be well recorded, particularly for chronic conditions,(15) there are some weaknesses with this approach. Low population coverage of the GPRD meant that only a small proportion of ALSPAC participants were captured through this linkage. Further, linked individuals only had a complete record while registered with a practice contributing to the GPRD. Having said this, out of 165 subjects in this study who ever had an asthma diagnosis, 65 (39%) were dated before their GPRD registration date. Also, high agreement between parent-reports and GP records suggest that missing historic diagnoses were not a substantial issue. A further shortcoming is the extent of missing ALSPAC data: complete data were only available for 29% of the linked subjects with known asthma status. If those without complete data were less likely to complete the questionnaires accurately than those with complete data then the levels of agreement presented here could be overestimates. However, the relative performance of the measures is unlikely to have been affected.

The main difference between this study and the majority of other asthma validation studies is that the symptom data in the latter were cross-sectional, so wheezing questions generally referred to the past 12 months and coincided with the timing of clinical assessment. These studies have generally concluded that wheezing questions are good at identifying asthmatics. A few studies have looked at "ever wheezing" to estimate the cumulative incidence of asthma. In these studies, the sensitivity and specificity of "ever wheezing" ranged from 80.6% to 94.5% and 74.9% to 89.9%, respectively.(5,17,18,19) However, in each case, this was based on a question asked at only one point in time. Jenkins et al found that reported wheezing was better at identifying adults than children with asthma and suggested that parents unfamiliar with wheezing may misreport other respiratory sounds as wheezing.(7) Asking the question, "Has your child ever wheezed?" at one time point is more likely to include children with persistent wheezing or asthma diagnosis due to difficulties of parental recall of sporadic wheeze in early childhood.(19) Because we asked questions about wheezing throughout early childhood, this may have included a higher proportion of subjects who

For peer review only - http://bmjopen.bmj.com/site/about/guidelines.xhtml

had viral infections causing wheezing. It has been estimated that 30-50% of preschool children experience episodic viral wheeze at least once.(20) This is supported by a Swedish study, where the prevalence of wheezing ranged from 26.6% among those aged 1-2 to 13.2% among those aged 5-6, whereas the prevalence of doctor-diagnosed asthma at these ages was 3.6% and 5.7%, respectively.(17) Further, results from another study showed that 48.5% of children aged 6 years had ever wheezed but 41% of these had transient early wheezing – at least one lower respiratory tract infection with wheezing before the age of 3 but no wheezing at 6 years; the majority of this group had no increased risk of asthma in later life(21). Our results regarding reported doctor-diagnosed asthma generally agree with previously published results. One study reported a sensitivity of 76.9% and a specificity of 97.5% for current doctor-diagnosed asthma, as determined from medical records.(17) Two recent studies of the cumulative incidence of childhood asthma determined from GP (UK-based) or health claims data (Canada) compared with parent-reported diagnosis reported high specificities but relatively low sensitivities.(22,23) Finally, de Marco et al found that the question "Have you ever had asthma?" agreed most closely with clinical diagnosis.(8)

Because of the lack of firm diagnostic criteria, the measurement of asthma in epidemiological studies is problematic. Using ever wheezing measured longitudinally from early childhood as a proxy for asthma is likely to greatly overestimate its prevalence, whereas reports of a doctor's diagnosis will estimate it reasonably accurately. Our results suggest that there is not an "ideal" way to define asthma in terms of wheezing alone. A measure which correctly identifies the majority of non-asthmatics will not pick up a very large proportion of children with asthma, and vice-versa. Having said this, the purpose of an epidemiological study is often to identify risk factors rather than to estimate prevalence. In general, relative risks will be biased towards the null in the presence of non-differential misclassification, and this is particularly dependent on a measure's specificity.(24) Pekkanen and Pearce suggest that a highly specific measure could be achieved by using a combination of BHR and symptoms or BHR, symptoms and reported doctor diagnosis.(3) In our

For peer review only - http://bmjopen.bmj.com/site/about/guidelines.xhtml

study, combining symptoms or BHR with a doctor's diagnosis certainly achieved high specificities. However, these combinations were not very sensitive and a doctor's diagnosis performed almost as well in terms of specificity but misclassified fewer asthmatics. Further, very high specificities were achieved by combining wheezing outcomes with reports of a doctor's diagnosis and these also misclassified fewer asthmatics. Peat et al suggest that using reports of a doctor's diagnosis is not good way to detect subjects with current severe asthma. They argue that identifying this group is important for determining risk factors and that this can be achieved with BHR.(25) Our data do not support this, as 65% of those with BHR had never received a diagnosis of asthma. Similarly, only 74% of those with BHR had ever wheezed.

In conclusion, we have found that parental reports of a doctor's diagnosis of asthma agree well with clinical records; we recommend that this question is incorporated into epidemiological questionnaires to supplement symptom data. The use of wheezing to define asthma is more problematic but our results suggest that reasonably high specificity can be achieved by using conditional questions about wheezing, although at the cost of lower sensitivity and failure to identify (probably) milder cases. We have also shown that linking data from observational studies to electronic patient records can be an effective means of validating parent-reported data, as well as providing a source of outcome data that may otherwise be missing. However, it should be acknowledged that the success of this methodology relies on good coverage of relevant datasets as well as low levels of dissent from participants to link to these.

ACKNOWLEDGEMENTS

We are extremely grateful to all the families who took part in this study, the midwives for their help in recruiting them, and the whole ALSPAC team, which includes interviewers, computer and laboratory technicians, clerical workers, research scientists, volunteers, managers, receptionists and nurses.

For peer review only - http://bmjopen.bmj.com/site/about/guidelines.xhtml

AUTHORS' CONTRIBUTIONS

RC carried out the statistical analysis and drafted the manuscript. JH contributed to the design of the study, interpretation of data and drafting of the paper. AB established the linkage process and contributed to the drafting of the paper. TVS established the linkage and data management process and reviewed the manuscript. RG cleaned, processed and advised on the ALSPAC asthma data and reviewed the manuscript. JM conceived the study and is PI of the Project to Enhance ALSPAC through Record Linkage (PEARL). All authors read and approved the final manuscript.

COMPETING INTERESTS

None

FUNDING

This work was supported by the Wellcome Trust (WT086118/Z/08/Z). The UK Medical Research Council (MRC), the Wellcome Trust and the University of Bristol currently provide core funding for ALSPAC (WT092731). Data collection is funded from a wide range of sources.

DATA SHARING

There are no additional data available.

For peer review only

REFERENCES

1. British Guideline on the Management of Asthma. *Thorax* 2008;**63**Suppl4:iv1-121 doi:10.1136/thx.2008.097741.
2. Global Initiative for Asthma. Global Strategy for Asthma Management and Prevention 2011. Available from: http://www.ginasthma.org/uploads/users/files/GINA_Report_2011.pdf. (accessed 5 Jun 2012)
3. Pekkanen J, Pearce N. Defining asthma in epidemiological studies. *Eur Respir J* 1999; **14**(4):951-7.
4. Asher MI, Keil U, Anderson HR et al. International Study of Asthma and Allergies in Childhood (ISAAC): rationale and methods. *Eur Respir J* 1995;**8**(3):483-91 doi:10.1183/09031936.95.08030483.
5. Sole D, Vanna AT, Yamada E et al. International Study of Asthma and Allergies in Childhood (ISAAC) written questionnaire: validation of the asthma component among Brazilian children. *J Investig Allergol Clin Immunol* 1998;**8**(6):376-82.
6. Remes ST, Pekkanen J, Remes K et al. In search of childhood asthma: questionnaire, tests of bronchial hyperresponsiveness, and clinical evaluation. *Thorax* 2002;**57**(2):120-6 doi:10.1136/thorax.57.2.120.
7. Jenkins MA, Clarke JR, Carlin JB et al. Validation of questionnaire and bronchial hyperresponsiveness against respiratory physician assessment in the diagnosis of asthma. *Int J Epidemiol* 1996;**25**(3):609-16 doi:10.1093/ije/25.3.609.
8. de Marco R, Cerveri I, Bugiani M et al. An undetected burden of asthma in Italy: the relationship between clinical and epidemiological diagnosis of asthma. *Eur Respir J* 1998;**11**(3):599-605 doi:10.1183/09031936.98.11030599.
9. Elphick HE, Ritson S, Rodgers H et al. When a "wheeze" is not a wheeze: acoustic analysis of breath sounds in infants. *Eur Respir J* 2000;**16**(4):593-7.

For peer review only

For peer review only - http://bmjopen.bmj.com/site/about/guidelines.xhtml

10. Lowe L, Murray CS, Martin L et al. Reported versus confirmed wheeze and lung function in early life. *Arch Dis Child* 2004;**89**(6):540-3 doi:10.1136/adc.2003.038539.

11. Toelle BG, Peat JK, Salome CM et al. Toward a definition of asthma for epidemiology. *Am Rev Respir Dis* 1992;**146**(3):633-7 doi:10.1164/ajrccm/146.3.633.

12. Boyd A, Golding J, Macleod J et al. Cohort Profile: The 'Children of the 90s'—the index offspring of the Avon Longitudinal Study of Parents and Children. *Int J Epidemiol* 2013;**42**(1): 111-127 doi:10.1093/ije/dys064 [published Online First: April 16, 2012].

13. Yan K, Salome C, Woolcock AJ. Rapid method for measurement of bronchial responsiveness. *Thorax* 1983;**38**(10):760-5 doi:10.1136/thx.38.10.760

14. Roberts G, Peckitt C, Northstone K et al. Relationship between aeroallergen and food allergen sensitization in childhood. *Clin Exp Allergy* 2005;**35**(7):933-940 doi:10.1111/j.1365-2222.2005.02280.x [published Online First: July 6th 2005].

15. Khan NF, Harrison SE, Rose PW. Validity of diagnostic coding within the General Practice Research Database: a systematic review. *Br J Gen Pract* 2010;**60**(572):e128-36 doi:10.3399/bjgp10X483562.

16. Mata Fernandez C, Fernandez-Benitez M, Perez Miranda M et al. Validation of the Spanish version of the Phase III ISAAC questionnaire on asthma. *J Investig Allergol Clin Immunol* 2005;**15**(3):201-10.

17. Hederos CA, Hasselgren M, Hedlin G et al. Comparison of clinically diagnosed asthma with parental assessment of children's asthma in a questionnaire. *Pediatr Allergy Immunol* 2007;**18**(2):135-41 doi: 10.1111/j.1399-3038.2006.00474.x [published Online First: 26 Oct 2006].

18. Lukrafka JL, Fuchs SC, Moreira LB et al. Performance of the ISAAC questionnaire to establish the prevalence of asthma in adolescents: a population-based study. *J Asthma* 2010;**47**(2):166-9 doi:10.3109/02770900903483766.

For peer review only - http://bmjopen.bmj.com/site/about/guidelines.xhtml

19. Luyt DK, Burton PR, Simpson H. Epidemiological study of wheeze, doctor diagnosed asthma, and cough in preschool children in Leicestershire. *BMJ* 1993;**306**(6889):1386-90 10.1136/bmj.306.6889.1386.

20. Townshend J, Hails S, McKean M. Diagnosis of asthma in children. *BMJ* 2007;**335**(7612):198-202 doi:10.1136/bmj.39234.651412.AE.

21. Martinez FD, Wright AL, Taussig LM et al. Asthma and Wheezing in the First Six Years of Life. *N Engl J Med* 1995;**332**(3):133-8 doi:10.1056/NEJM199501193320301.

22. Yang CL, To T, Foty RG et al. Verifying a questionnaire diagnosis of asthma in children using health claims data. *BMC Pulm Med* 2011;11:52 doi:10.1186/1471-2466-11-52.

23. Canova C, Harris JM, Mills P et al. Epidemiological measures of childhood asthma: Cross-sectional and longitudinal consistency. *Respir Med* 2012;**106**(9):1226-1235 doi:10.1016/j.rmed.2012.05.008 [published Online First: 16 June 2012].

24. Copeland KT, Checkoway H, McMichael AJ et al. Bias due to misclassification in the estimation of relative risk. *Am J Epidemiol* 1977;**105**(5):488-95.

25. Peat JK, Toelle BG, Marks GB et al. Continuing the debate about measuring asthma in population studies. *Thorax* 2001;**56**(5):406-11 doi:10.1136/thorax.56.5.406.

Licence statement

The Corresponding Author has the right to grant on behalf of all authors and does grant on behalf of all authors, an exclusive licence (or non-exclusive for government employees) on a worldwide basis to the BMJ Group and co-owners or contracting owning societies (where published by the BMJ Group on their behalf), and its Licensees to permit this article (if accepted) to be published in any BMJ Group products and to exploit all subsidiary rights, as set out in our licence.

For peer review only
